# Financial ratios, corporate governance and bank-firm information: a Bayesian approach to predict SMEs’ default

**DOI:** 10.1007/s10997-021-09614-5

**Published:** 2022-02-17

**Authors:** Carmen Gallucci, Rosalia Santullli, Michele Modina, Vincenzo Formisano

**Affiliations:** 1 Department of Management and Innovation Systems, University of Salerno, IPAG Business School, Paris, France; 2 Department of Economics, University of Genoa, Genova, Italy; 3 IPAG Business School, Paris, France; 4 University of Molise, Campobasso, CB Italy; 5 University of Cassino and Southern Lazio, Cassino FR, Italy

**Keywords:** Default risk, Financial distress, Small and medium-sized enterprises, Bayesian methodology

## Abstract

The paper aims to jointly combine three different categories of variables (financial ratios, corporate governance data and bank-firm information) to predict SMEs’ default. To this end, a merged longitudinal predictive model was applied to a sample of 973 Italian SMEs that are clients of 36 different co-operative banks. The collected data (for a total of 23 variables included in the model) relate to the years 2012–2014. The main findings reveal the high predictive power of leverage ratio, CEO tenure and ownership concentration, and the number of loans overdue for more than 180 days in the previous 12 months.

## Introduction

The issue of default-prediction models for small and medium-sized enterprises (SMEs) has attracted the interest of academics since the 1970s (Edmister, 1972), with renewed attention starting from the 1990s due to the Basel Capital Accords. The unique financial characteristics of SMEs have meant that traditional default-prediction models (developed for large firms) are not adequate for estimating the probability of SMEs defaulting (Ciampi, 2015). SMEs are actually financially riskier and have a lower asset correlation with one another than large businesses (Dietsch & Petey, 2004; Saurina & Trucharte, 2004). As a consequence, the development of default-prediction models for SMEs has become a specific and autonomous stream of finance literature, and this is still the case today.

This field of interest has become particularly topical given the current COVID-19 pandemic, which is making the limits of traditional rating models (mainly based on financial ratios and accounting data) even more marked when applied to SME default prediction. The global COVID-19 crisis has been impacting on the financial health of the majority of SMEs, forcing them to base their chances of survival on turnaround plans which, by their very nature, significantly reduce the predictive value of past accounting data on which financial ratios are based (Ciampi et al., 2021). Furthermore, this crisis is expected to have an amplification effect on the tendency of SMEs to resort to unorthodox accounting behaviors with the aim of postponing the emergence of economic and/or financial imbalances (Ciampi, 2018), thereby increasing the timeframe within which a firm’s financial weaknesses are reflected inits financial ratios level.

It is, then, necessary to build default-prediction models tailored to SMEs (Calabrese et al., 2013) and based on other information, in addition to traditional financial ratios (Ciampi et al., 2021). Some studies (among others, Ohlson, 1980; Keasey & Watson, 1987; Lin et al., 2010; Ciampi, 2015; Christopoulos et al., 2019) have, for example, already analyzed the effect of corporate governance characteristics on SEM failure by demonstrating that adding corporate governance variables to financial ratios significantly improves SME default-prediction accuracy rates. Other studies (among others, Bartoli et al., 2013; Bergerès et al., 2015; Chen et al., 2015; Norden & Weber, 2010; Smondel, 2018) have instead considered data related to the relationship with banks, suggesting that also adding this information improves the predictive power of models. However, to date, no studies have combined corporate governance characteristics and bank-firm information together with financial ratios, while errors in prediction models proposed in the literature still persist. The challenge is finding a prediction system based on a consistent number of different variables (Ciampi, 2015, 2021).

An appropriate methodology to combine financial, corporate governance and bank-firm data can be identified in Bayesian models (Bernardo & Smith, 1994), which increase in-sample prediction accuracy in comparison to traditional logit models (Figini & Giudici, 2011). This methodological approach was adopted for the present study, specifically by running a merged longitudinal predictive model on a sample of 973 SMEs based in Italy, where small and medium-sized enterprises account for more than 98% of all firms and employ over 80% of the total workforce. The firms in the sample are clients of 36 different co-operative banks (7.6% of the banks operating in Italy). They have a direct presence in more than a third of Italian municipalities, and in 620 municipalities (out of 7903), they operate as a single intermediary (Bank of Italy, 2019). Their commitment to retail banking is evidenced by the fact that 59% of their assets are destined for loans to households and small and medium-sized businesses (6% more than other banks). The data were collected for the years 2012–2014.

The main findings suggest the high predictive power of leverage ratio (total debts/(total debts + equity)), CEO tenure, ownership concentration and board diversity, and the number of loans overdue for more than 180 days in the previous 12 months and the number of months during which enterprises are overdrawn – their cash and signature during the previous 12 months.

The paper contributes to the stream of finance literature on SME default prediction by drawing upon a combination of the following: financial ratios (cash flow/turnover, cash flow/total debts, return on investment, acid test ratio and total debts/(total debts + equity)); interest charges/turnover; EBITDA/turnover; total debts/EBITDA; corporate governance data (CEO duality, board independence, CEO tenure, ownership concentration, board size, equity incentives of the board, board diversity and audit independence); and bank-firm information (reporting institutions – 12 months; first information requests – 12 months; suffering – 12 months; overdue 180 days – 12 months; revocation – 12 months; expense on used – 12 months; months overdrawn). A close examination of published literature (as reported in the next section) highlights that although these three categories have been considered in previous studies analyzing their predictive power in relation to SME defaults, such studies have never combined all three categories. By doing this, we have answered the call for exploration and measurement of the predictive power of different variables and/or categories of variables, emerging from the systematic literature review on SME default prediction by Ciampi et al. (2021). Furthermore, to fully capture the predictive power of the three different categories of variables, we applied a merged longitudinal predictive model (Figini & Giudici, 2011), anchored in the Bayesian literature (Bernardo & Smith, 1994), which performs much better than classical longitudinal models and logit models (Figini & Giudici, 2011). Lastly, by using data from several banks, we had the opportunity to control our analysis for bank characteristics.

## SME default-prediction modeling: a review of the literature

The academic literature on financial distress forecasting dates back to the work of Beaver (1966), who first tested the predictive ability of a selection of financial ratios by conducting a univariate analysis of a matched-pair sample of large US firms. The topic then gained momentum thanks to contributions from several prominent authors who estimated the likelihood of large firms incurring financial distress, using approaches such as multiple discriminant analysis (Altman, 1968), methods for pricing corporate liabilities (Merton, 1974) and logistic and probit regressions (Ohlson, 1980; Zmijewski, 1984). However, it was only with the seminal investigation of Edmister (1972) that research into default-prediction models specific to SMEs started attracting scholarly attention. Edmister (1972) selected 42 US SMEs and 19 financial ratios and, like Altman (1968), relied on multiple discriminant analysis. He concluded that the application of such a technique results in improved predictions, compared to traditional subjective evaluations.

From the mid-1980s onwards, scholars primarily relied on logistic and probit regressions to predict SME defaults. Keasey and Watson (1987) focused on single-plant, independently owned SMEs located in the northeast of England between 1970 and 1983 and developed one of the first logit models for financial distress prediction, based on accounting and firm-level data. Apart from the UK (Gupta et al., 2015; Lin et al., 2012), similar single-country empirical analyses relying on accounting and firm-level data were later conducted for SMEs in several different geographical contexts, such as Sweden (Yazdanfar, 2011), Greece (Kosmidis & Stavropoulos, 2014) and Lithuania (Kanapickiene & Spicas, 2019). Instead, probit regression, first proposed by Zmijewski (1984), grew in importance as a research method in the early 2000s, when Dietsch and Petey (2002) conducted a large-scale study on a sample of 220,000 French SMEs, employing predominantly accounting and credit scoring data. In the following years, researchers also started to use loan-level data collected from the internal databases of banks operating in Germany (Norden & Weber, 2010), Slovakia (Fidrmuc & Hainz, 2010), Italy (Bottazzi et al., 2011) and Portugal (Duarte et al., 2018). However, although regression models can ensure the transparency of results when examining SMEs’ likelihood of incurring financial failure, these models suffer from high sensitivity to multicollinearity, and data should be coherent with the statistical assumptions that these models imply. Furthermore, although their accuracy is satisfactory, regression models are still limited compared to non-parametric models (Alaka et al., 2018).

In the late 1980s, Bayesian approaches were developed to predict financial failure of SMEs. These models were primarily based on accounting data and presented lower loss functions compared to their traditional counterparts. In their pioneering work, Keasey and Watson (1988) examined a matched-pair sample of 73 failed SMEs located in the northeast of England between 1970 and 1982 and found that a financial failure-prediction model based on a simple Bayesian approach correctly classified 69.9% of non-failed and 65.8% of failed SMEs. In the following years, Fantazzini and Figini (2009) observed a panel of 1003 German SMEs belonging to 352 business sectors between 1996 and 2004 and obtained evidence that Bayesian models outperform classical longitudinal and pooled logit models. Recently, Traczynski (2017) proposed a Bayesian model incorporating market data, which could provide better out-of-sample forecasts using fewer, albeit significant, predictors. Although they combine simplicity, interpretability and predictive accuracy, Bayesian models are highly dependent on the availability of prior knowledge. Therefore, having sufficiently large datasets is a critical issue (Fantazzini & Figini, 2009).

From the early 2000s onwards, credit scoring and hazard models entered the academic debate on SME default prediction. Credit scoring models brought several relevant methodological improvements. In this regard, Sohn and Kim (2013) built a model that considers four different states of financial ratios, while Nam (2013) devised a forecasting tool that captures (month by month) the countercyclical movements of capital requirements for SMEs. More recently, Chi and Meng (2019) proposed a rating system for SMEs that avoids information redundancy by relying on a small selection of 23 indices deriving from accounting-, firm-, loan- and market-level data. Credit scoring models, albeit superior in forecasting accuracy compared to more traditional empirical approaches, usually depend on the availability of several categories of data (e.g., Hasumi & Hirata, 2014; Sohn & Jeon, 2010; Sohn & Kim, 2013; Yu et al., 2019), and their classification performance may be undermined by the subjective biases of the human raters involved (Oliveira et al., 2017). Hazard models have also proved effective in predicting SMEs’ failure, thanks to their ability to deal with time-varying covariates (El Kalak & Hudson, 2016; Gupta et al., 2018). Nevertheless, they require compliance with statistical assumptions concerning the data structure and management of missing data.

Over the last decade, to overcome the main limitation inherent in traditional models, artificial intelligence tools have been developed to predict the financial failure of SMEs. Angelini et al. (2008) first proposed a feed-forward and an ad-hoc artificial neural network to assess the credit risk of 76 Italian SMEs, based on accounting and loan-level data, reporting error rates ranging from 7% to 14%. Mittal et al. (2011) also built a non-parametric multilevel perceptron to assess the credit reliability of a sample of 2864 Indian micro enterprises observed from 2007 to 2009, presenting an overall predictive accuracy of 71.68%. Ciampi and Gordini (2013) analyzed over 7000 Italian SMEs and found that artificial neural networks outperformed multiple discriminant analysis and logistic regression regardless of the level of aggregation considered (size, business sector and geography). Besides being very accurate non-parametric tools, artificial neural networks benefit from low sensitivity to multicollinearity. However, the results they provide lack transparency, causing researchers to refer to artificial neural networks as “black boxes” (Alaka et al., 2018).

Overall, extant studies on default prediction of SMEs have relied on parametric models implying strong statistical assumptions in the data structure (e.g., Gupta et al., 2015; Lin et al., 2012) or non-parametric models lacking transparency (Angelini et al., 2008; Ciampi & Gordini, 2013; Mittal et al., 2011). Moreover, even though accounting-, firm- and loan-level data have been used separately in previous analyses, their joint use is still limited (e.g., Yildirim, 2020). Furthermore, corporate governance data have seldom been included in forecasting models, despite their utmost relevance in the context of SMEs (Ciampi, 2015). When incorporated in quantitative models, corporate governance data have been fed into traditional regression models (Filipe et al., 2016; Keasey & Watson, 1987; Ohlson, 1980; Ono et al., 2014).

The database used in the present paper, being sufficiently large, facilitated use of a Bayesian classifier, thus overcoming the limitations inherent in both traditional and artificial intelligence models. Moreover, the empirical model proposed employs accounting, corporate governance and bank-firm data, thus encompassing all classes of predictors that the extant literature has deemed mandatory for forecasting the financial failure of SMEs.

## Methodology

### Sample and data collection

The analysis was run on a sample of 973 Italian small and medium-sized enterprises, which are clients of 36 different co-operative banks. The initial dataset was provided by Centrale Rischi Finanziari (CRIF: Italian Credit Register), an Italian rating agency, Centro Servizi Direzionali (CSD), an Italian consulting company working with the co-operative banks, and Cerved, a data-driven company that holds information about governance and ownership structures, and copies of the balance sheets and income statements of all Italian companies. After excluding large firms from the initial sample, we were left with 1847 small and medium-sized enterprises. To classify the small and medium-sized enterprises, we referred to the European definition that considers a firm as an SME if it has fewer than 250 employees and a total revenue below 50 million euro. In the second step, we excluded firms that did not have three years (2012–2014) of complete information, resulting in a sample of 1771 firms, which was split into small and medium-sized enterprises that had defaulted or not defaulted. We referred to the new definition of “default” (Article 178 of Regulation (EU) No. 575/2013 (Capital Requirements Regulation – CRR)), which came into force in Italy on January 1, 2021. With regard to this article, the European Banking Authority (EBA) guidelines specify all aspects relating to application of the definition of default of an obligor. The EBA has identified differing practices used by institutions concerning the definition of default. Consequently, these guidelines provide detailed clarification of application of the definition of default, which includes aspects such as the following: “days past due” criterion for default identification; indications of unlikeliness to pay; conditions for a return to non-defaulted status; treatment of the definition of default in external data; application of the default definition in a banking group; and specific aspects related to retail exposures. The EBA considers this harmonization necessary in order to ensure consistent use of the definition of default and to ensure that a harmonized approach is taken across institutions and jurisdictions. By applying this definition, we identified 252 defaulted firms.

We then used a propensity score-matching method to identify a sample of comparable firms with similar characteristics as defaulted or non-defaulted. In order to identify a set of companies which did not differ significantly from the sample of defaulted firms in specific criteria, we firstly split the whole sample into age-location-industry-dimension (employees) subsamples, and we ran separate propensity score logit regressions for each subsample. By matching defaulted firms to controls in the same place and industry, of the same age and dimension, year by year, we mitigated concerns that a non-random age/location/industry/dimension distribution could affect the results. We applied three-to-one nearest-neighbor matching with replacement. In detail, we identified the matching partners for each defaulted firm by minimizing the propensity score distance between defaulted and non-defaulted firms. In order to meaningfully employ matching, it was necessary to condition the support common to both defaulted and non-defaulted firms (Heckman et al., 1998). Implementing the common support condition ensured that any combination of characteristics observed in the defaulted group could also be observed in the control group. We employed the minima and maxima comparison method and deleted all observations where the propensity score was smaller than the minimum and/or larger than the maximum in the opposite group (see Caliendo & Kopeinig, 2008). In addition, we checked whether the matching procedure was able to balance distribution of the relevant variables in both the control and buyout groups (“balancing property”). Our final sample included 721 control firms, with a total of 973 firms.

This procedure, by controlling for constant unobserved differences between the treatment and the control group, allowed us to reduce self-selection problems and avoid the existence of systematic differences in distribution of the matched samples after the matching process (Rosenbaum & Rubin, 1983).

The resultant sample of 973 SMEs included firms from the manufacturing industry (27%), the service industry (46%) and others (27%), but not financial companies. It was composed as follows: 603 micro enterprises (turnover < 2 mln; employees < 10); 292 small enterprises (turnover < 10 mln; employees < 50); 78 medium-sized enterprises (turnover < 50 mln; employees < 250). The firms in our sample are mainly located in the north and centre of Italy and have the legal form of a limited company (89%) (Table 1).

**Table 1:** Sample distribution

	Total sample (%)	Non-default firms (%)	Default firms(%)
*Industry*			
Manufacturing	27	20	21
Service	46	50	64
Others	27	30	15
*Size*			
Micro	62	73	57
Small	30	24	34
Medium	8	3	9
*Location*			
North	78	78	79
Centre	18	20	16
South and Islands	4	2	5
*Legal form*			
Limited companies	89	72	57
No limited companies	11	28	43

As anticipated, the firms in the sample are clients of 36 different co-operative banks, with capital of between 5 thousand euro and 322 thousand euro, and total assets between 44 thousand euro and about 8 and a half million euro. In Italy, co-operative credit is the most representative form of banking localism. The small size and orientation towards the local market favor relationship lending and reduction of information asymmetries between lenders and borrowers (Berger & Udell, 1995; Berger et al., 2005; Elsas, 2005; Petersen & Rajan, 1994). The information advantage enjoyed by co-operative banks over their larger counterparts, as well as their proximity to the entrepreneurial and social fabric of the territory, translates into a better capacity to select and monitor opaque borrowers such as SMEs (McKillop et al., 2020). Compared to local banks established in another corporate form, co-operative credit banks have similar purposes, adopt a traditional banking business model and use homogeneous organizational structures and procedures, which are evidenced by recent formation of co-operative banking groups (Iccrea and Cassa Centrale Banca). This aspect contributes to the originality of our data because we could also capture bank-level heterogeneity, and it was possible to extend the results to a greater part of the Italian banking system than most previous studies focusing on single banks or groups of banks (e.g., Dainelli et al., 2013).

For each firm in our sample, Cerved provided data about financial statements and governance and ownership structures; CRIF and CSD made bank-firm hard information available. Finally, data about banks were collected through the Bankscope Bureau Van Dijk database. The years covered by the analyses were 2012, 2013 and 2014; and the total number of observations was 2147.

### Variables

This study used one dependent variable and three groups of independent variables: financial ratios, bank-firm information and corporate governance, plus some control variables at bank and firm level. More details are provided below.

Dependent variable: This was the *default risk*, which was measured by a dichotomous variable (*probability of default*), taking a value of 1 if a firm had gone bankrupt or defaulted and 0 otherwise.

Independent variables: As stated above, this study used three groups of independent variables: financial ratios, bank-firm hard information and corporate governance indicators (Table 2).

**Table 2:** List of financial ratios

Profitability	Leverage	Liquidity
Cash flow/turnover^*^	Value added/turnover	Long term assets/number of employees
Return on equity	Bank loans/turnover	Current ratio
Interest charges/bank loans	Net financial position/turnover	Cash flow/total debts^*^
Return on investment^*^	EBITDA/cash flow	Acid test ratio^*^
Turnover/net operative assets	Total debts/(total debts + equity)^*^	
Return on sales	Interest charges/turnover^*^	
EBITDA/turnover^*^	Financial debts/equity	
	Interest charges/EBITDA	
	Total debts/EBITDA^*^	
	Turnover/number of employees	
	Equity/long-term	
	Value added/number of employees	

^*^Financial ratios selected after applying VIF procedure and stepwise method

With regard to financial ratios, we included ratios for profitability, leverage and liquidity areas of a company’s profile. Specifically, by applying the variance inflation factor (VIF) method (Chatterjee & Hadi, 2012), we moved from a list of 23 ratios previously used in the literature on SME default prediction (Ciampi, 2015) and selected those with the lowest rates of correlation, excluding variables with VIF values above 3 (Pompe & Bilderbeek, 2005). Finally, to identify the best combination of these, we applied the stepwise method (Shin & Lee, 2002; Shin et al., 2005), resulting in seven ratios for inclusion in the regression models.

Bank-firm information was selected from a long list of 365 variables provided by CRIF and CSD, by following the same procedure explained above (variance inflation factor and stepwise methods). Table 3 shows the seven variables selected and their measures.

**Table 3:** Bank-firm hard information

Variables	Measures
Reporting institutions—12 month	The average number of reporting institutions in the last 12 month
First information requests—12 months	The average number of first information requests in the last 12 months
Suffering—12 months	The presence of suffering in the last 12 months
Overdue 180—12 months	The number of loans with overdue 180 in the last 12 months, used upon agreement
Revocation—12 months	The number of revocation in the last 12 months
Expense on used—12 months	The amount of expense on used—self-liquidating in the last 12 months
Months with overdraft	The number of months with overdraft—cash and signature in the last 12 months

Finally, the corporate governance variables were also selected, firstly by looking at previous literature on the corporate governance-default relationship and then by applying the variance inflation factor and stepwise methods. The selected variables and their measures are listed in Table 4.

**Table 4:** Corporate governance indicators

Variables	Measures
CEO-duality	This variable assumes value 1 for firms in which the CEO is also the chair of the board of directors and value when the CEO and the chair of the board are two distinct people
Board independence	This variable is measured by the percentage of directors who do not have any affiliations with the firm
CEO tenure	It is measured by the number of years since a CEO has been serving on the board
Ownership concentration	This variable assumes value 1 for firms in which a shareholder (or more shareholders belonging to the same family) holds (hold) the majority of the shares, 0 otherwise
Board size	It is operationalized as the number of directors on the board
Equity incentives of the board	It is measured by the percentage of shareholdings owned by all the directors
Board diversity	This variable is measured as the percentage of female directors on the board
Audit independence	This variable is measured as the percentage of independent directors on the audit committee

Control variables: We included the following control variables: *firm size*, *industry*, *location*, *firm age*, *legal form* and *bank capitalization*. *Firm size* was measured as the natural logarithm of the number of employees. The entire sample of examined firms consisted of SMEs. However, as reported in the sample description, these could be split into micro, small and medium-sized enterprises. It is thus possible for different turnover levels to influence the default probability. *Industry* was considered through two dummy variables relating to the sectors to which the firms belonged: manufacturing and services (with “other industries” as the reference category). This partition, which is generally used by Italian banks to develop their scoring and rating systems, allowed us to capture the possible effects of typically diverse profiles of firms operating in the three different categories of business, in terms of financial and governance structures. *Location* was operationalized through three dummy variables concerning the geographical location of the firms: north, center and south/islands of Italy (with “centre” as the reference category); this geographical partitioning is frequently used in research on Italian samples and allowed us to capture different economic and industrial features characterizing the Italian business system. *Firm age*, which is strictly linked to firms’ probability of default (as previous studies have suggested), was measured as the natural logarithm of the years since constitution. *Legal form* was a dummy variable assuming a value of 1 when the firm was a limited company and 0 otherwise. Finally, at bank level, bank capitalization was included in the regression models, measured as CET 1 (common equity tier 1), calculated as the TIER1/asset ratio.

### The merged longitudinal predictive model

The model applied to study the joint effect of financial ratios, corporate governance variables and bank-firm information was a merged longitudinal predictive model, which performs better than both separate longitudinal predictive models and a single model (Figini & Giudici, 2011). The combined model finds justification in the Bayesian paradigm, which provides a unified and intuitively appealing approach to the problem of drawing inferences from observations. Bayesian statistics view statistical inference as a problem of belief dynamics, and evidence about a phenomenon is used to revise and update the knowledge about it. Following Bayesian theory (see, e.g., Bernardo & Smith, 1994) and the approach by Figini and Giudici (2011), we firstly observed a score π_i_ i = 1,.., n derived from financial ratios; a score π_i_^*^ i = 1,.., n derived from corporate governance data; and a score π_i_^**^ i = 1,.., n derived from bank-firm information. Secondly, starting from the original training data D of size n (composed of k financial variables, z corporate governance variables and p bank-firm variables), we generated r new training sets by sampling examples from D uniformly and with replacement. As a result, we obtained r bootstrapped training sets. Thirdly, we built r predictive models on the r bootstrapped training datasets derived from step 1 separately for the financial ratios, corporate governance and bank-firm variables. In the fourth step, we computed the variances of π_I_, π_i_^*^ and π_i_^**^. In the fifth step, we derived δ_i_ as

$$\mathrm{\delta i }=\frac{\frac{1}{{\sigma }^{2}({\pi }_{i})}}{\frac{1}{{\sigma }^{2}({\pi }_{i})}+ \frac{1}{{\sigma }^{2}({\pi *}_{i})}+ \frac{1}{{\sigma }^{2}({\pi **}_{i})}}$$

The final probability of default for each SME was a linear combination of π_I_, π_i_^*^ and π_i_^**^ weighted by δ_i_, computed as follows:

$${\text{PD}}_{\text{i}} = \updelta_{\text{i}}\uppi_{\text{I}} + (1 - \updelta _{\text{i}})\uppi_{\text{i}}^{*} + (1 - \updelta _{\text{i}})\uppi_{\text{i}}^{**}, \quad {\text{i}} =1, \ldots , {\text{n}}$$

It can be interpreted as a Bayesian before posterior probability for the models proposed. It should be noted that the data indicator δ_i_ had to satisfy suitable regularity conditions: σ^2^ (π_i_^**^) + σ^2^ (π_i_^*^) + σ^2^(π_i_) ≠ 0, σ^2^ (π_i_^**^) < ∞, σ^2^ (π_i_^*^) < ∞, and σ^2^ (π_i_) < ∞.

By following Figini and Giudici (2011), we further estimated the posterior expectation of θ, as follows:

$${\text{E}}(\theta /{\text{y}}){\text{ }} = \frac{{k~ + ~a_{0} }}{{n + ~a_{0} + \beta _{0} }} = ~\frac{n}{{n + ~a_{0} + \beta _{0} }}~.~\frac{k}{n} + \frac{{a_{0} ~ + \beta _{0} }}{{n + ~a_{0} + \beta _{0} }}~\left( {\frac{{a_{0} }}{{a_{0} ~ + \beta _{0} }}} \right)$$

## Results

### Descriptive statistics

Looking at financial ratios, we could note the sign was minus before the mean value of return on investment and EBITDA/turnover. This indicates that the levels of profitability for the firms in our sample were negative on average. The degree of indebtedness, on the other hand, was almost as high as the level of liquidity (taking into account the inventory).

The CEO was also chair of the board of directors in less than 50% of the firms in the sample; 20% of directors were not officers and did not have any affiliations with the firm. The maximum number of years that a CEO had served on the board was 37, and the median was 9. In 80% of the firms, a shareholder (or more shareholders belonging to the same family) held the majority of the shares. The number of directors on the board was equal to 3.19 on average, with a maximum value of 25. Women constituted 22% of the directors on average; the mean percentage of independent directors on the audit committee was 29%.

By splitting the sample between default and non-default firms, we could observe that although it was negative for both default and non-default firms, the return on investments was, on average, significantly lower for default firms. Moreover, firms that were more indebted also presented a lower degree of liquidity. Additionally, default firms presented a higher number of credit-limit violation days on a checking account in a month and a higher value of overdue payments at the end of the month. Finally, default firms were smaller than non-default ones (Table 5).

**Table 5:** Descriptive statistics

Variables	Mean	Median	SD	Min	Max
*Dependent variables*					
Probability of default	0.5	0.5	0.5	0	1
Financial distress	1.91	0.84	5.54	−5.18	80.60
*Independent variables*					
*Financial ratios*					
Return on investment	−7.47	1.4	38.71	−405.78	45.87
EBITDA/turnover	−0.18	0.05	1.99	−47.63	0.84
Interest charges/turnover	0.07	0.03	0.42	0	10.18
Total debts/(total debts + equity)	0.92	0.83	0.65	0.10	8.72
Total debts/EBITDA	10.07	6.82	185.12	−2529.47	3624.61
Acid test ratio	71.86	66.72	33.83	2.68	242.98
Cash flow/total debts	−0.00	0.02	0.24	−1.05	2.69
*Bank-firm hard information*					
Reporting institutions—12 month	2.8	2.7	0.31	0	5
First information requests—12 months	1.7	2.1	1.03	0	3
Suffering—12 months	45.76	48.72	12.01	0	73.15
Overdue 180—12 months	37.1	36.6	5.09	0	88
Revocation—12 months	1.84	1.93	0.87	0	4
Expense on used—12 months	15.76	12.48	8.04	0	22.56
Months with overdraft	4.2	4.7	2.13	0	6
*Corporate governance indicators*					
CEO-duality	0.04	0	0.20	0	1
Board independence	0.20	0.16	0.19	0	1
CEO tenure	13.60	9	17.42	0	37
Ownership concentration	0.80	1	0.40	0	1
Board size	3.19	2	2.99	0	25
Equity incentives of the board	0.58	0.47	0.39	0	1
Board diversity	0.22	0	0.29	0	1
Audit independence	0.29	0.19	0.19	0	0.60
*Control variables*					
Firm size	17.30	7	29.93	0	249
Manufacturing	0.21	0	0.39	0	1
Service	0.58	1	0.49	0	1
North	0.78	1	0.28	0	1
South/Islands	0.04	0	0.19	0	1
Legal form	0.66	1	0.47	0	1
Firm age	18.03	15	12.10	1	73
Bank capitalization	15.68	14.89	5.31	12.02	20.28

In terms of the corporate governance indicator, it was possible to affirm that in default firms, the CEO was generally not also chair of the board of directors, and he/she tended to lead the firm for a shorter period (5.3 years on average) than CEOs of non-default firms (26.5 years on average); the degree of ownership concentration was slightly higher, while the board size was smaller; finally, there was a significantly lower percentage of women on the board.

Table 6 shows the pairwise correlations, where we can observe that while the return on investments, the EBITDA/turnover ratio and the acid test were negatively correlated to default risk, the total debts/(total debts + equity) ratio and the total debts/EBITDA ratio were significantly and positively correlated. CEO duality, CEO tenure, board independence and board diversity were significantly and negatively correlated with SME default, while ownership concentration was significantly and positively correlated to default risk. Finally, the number of months that firms were overdrawn for, and overdue payments, were both significantly and positively related to SME defaults.

**Table 6:** Descriptive statistics for default and non-default firms

Variables	Non-default	Default	T-test
	Mean	Mean	
*Financial ratios*			
Return on investment	−0.78	−22.38	14.61**
EBITDA/turnover	−0.12	−0.31	1.72
Interest charges/turnover	0.03	0.16	1.84
Total debts/(total debts + equity)	0.76	1.27	0.89***
Total debts/EBITDA	17.62	−6.79	11.52
Acid test ratio	73.36	52.10	15.67**
Cash flow/total debts	0.047	−0.12	−1.31
*Bank-firm hard information*			
Reporting institutions—12 month	0.4	3.9	2.41
First information requests—12 months	0.78	1.73	6.21
Suffering—12 months	18.8	52.7	11.74
Overdue 180—12 months	0.84	2.83	5.64***
Revocation—12 months	8.07	21.76	5.76
Expense on used—12 months	2.7	5.8	1.48
Months with overdraft	1.7	4.89	10.35***
*Corporate governance indicators*			
CEO-duality	0.07	0.00	1.23**
Board independence	0.36	0.15	2.15
CEO tenure	26.5	5.3	12.54*
Ownership concentration	0.77	0.83	7.89*
Board size	3.69	2.5	10.43**
Equity incentives of the board	0.64	0.56	9.67
Board diversity	25.80	17.37	11.23***
Audit independence	0.32	0.19	7.45
*Control variables*			
Firm size	20.83	12.46	13.45**
Manufacturing	0.20	0.21	4.56
Service	0.5	0.68	3.89
North	0.78	0.79	10.79
South/Islands	0.02	0.05	9.67
Legal form	0.72	0.57	5.87
Firm age	17.67	18.51	11.98

*p < .0.10; **p < 0.05; ***p < 0.01

### Results of merged longitudinal predictive model

Table 7 shows the results obtained by applying a longitudinal predictive model to estimate the predictive power of financial ratios.

**Table 7:** Chosen financial ratios for one step LPM

Variables	Estimate	Std. Error	*p*-value
(intercept)	0.542	1.62	<0.0001
Total debts/(total debts + equity)	3.561	2.12	<0.0001
Total debts/EBITDA	2.314	1.43	<0.0001

The results suggest that the degree of indebtedness is significantly and positively related to default risk, thus suggesting that because more indebted SMEs have a higher probability of failing, choices about firms’ financial structure cannot be undervalued.

Table 8 shows the results obtained by applying a longitudinal predictive model to estimate the predictive power of corporate governance variables.

**Table 8:** Chosen corporate governance variables for one step LPM

Variables	Estimate	Std. Error	*p*-value
(intercept)	0.411	0.78	<0.0001
CEO tenure	−4.21	1.44	<0.0001
Ownership concentration	0.74	0.98	<0.0001
Board diversity	−2.103	1.01	<0.0001

We found that CEO tenure is negatively related to SME defaults. This means that when a CEO sits on the board for a long time, the probability of a default is reduced. Again, ownership concentration is positively related to the probability of default. This means that for SMEs, the presence of a majority shareholder is not a guarantee of the firm’s continuity. Furthermore, the probability of default is affected by board diversity, in such a way that a higher percentage of women on the board reduces the default risk.

Table 9 shows the results obtained by applying a longitudinal predictive model to estimate the predictive power of bank-firm hard information.

**Table 9:** Chosen bank-firm hard information for one step LPM

Variables	Estimate	Std. Error	*p*-value
(Intercept)	0.228	1.04	<0.0001
Overdue 180–12 months	0.368	0.54	<0.0001
Months with overdraft	0.102	0.73	<0.0001

The number of loans overdue by 180 days in the previous 12 months and the number of months overdrawn – cash and signature in the preceding 12 months – significantly affect the probability of default.

Table 10 shows the results for a single model, with a significant just leverage ratio, CEO tenure and ownership concentration, and number of loans overdue by more than 180 days in the last 12 months.

**Table 10:** Chosen predictors for one step LPM

Variables	Estimate	Std. Error	*p*-value
(Intercept)	0.478	1.12	<0.0001
Total debts/(total debts + equity)	3.542	2.01	<0.0001
CEO tenure	−4.18	0.43	<0.0001
Ownership concentration	0.63	0.98	<0.0001
Overdue 180–12 months	0.322	1.05	<0.0001

### Robustness check

To corroborate the robustness of our results, we also used an alternative and continuous measure of default risk: *financial distress* (Migliani et al., 2015). The measure for this variable was based on the model developed by Zmijewski (1984). The Zmijewski Financial Score (ZFS) is one of the most widely used financial distress-prediction models (Carcello & Neal, 2003; Hay et al., 2007). The ZFS is constructed based on an index incorporating multiple financial ratios representing firm profitability (net income/total assets), leverage (total debt/total assets) and liquidity (current assets/current liabilities). A higher ZFS indicates a greater likelihood of financial distress.

We ran a merged longitudinal predictive model for this dependent variable. Table 11 shows the results and confirms the robustness of our analysis.

**Table 11:** Chosen predictors for one step LPM (Financial distress)

Variables	Estimate	Std. Error	*p*-value
(Intercept)	0.478	1.12	<0.0001
Total debts/(total debts + equity)	3.211	1.89	<0.0001
CEO tenure	−3.79	0.79	<0.0001
Ownership concentration	0.82	0.47	<0.0001
Overdue 180–12 months	0.274	1.21	<0.0001

## Concluding discussion

The paper aimed to examine the joint effect of financial ratios, corporate governance and bank-firm information on SME defaults, by applying a merged longitudinal predictive model (Figini & Giudici, 2011) anchored in Bayesian literature (Bernardo & Smith, 1994). This served to overcome the difficulties found in previous studies of keeping a huge amount of information for SMEs, given their reduced dimension and the reluctance of banks to share credit relationship data (Altman et al., 2013).

Our main results relating to financial ratios suggest that leverage is positively related to SMEs’ default risk. Therefore, we suggest that firms pay attention to their level of indebtedness and also to the structure of their debt. Of particular interest are our findings about the effect of corporate governance variables on SMEs’ default risk. Specifically, the results show that CEO tenure has a significant and negative effect on the probability of default, meaning that when a CEO serves on the board for a long period of time, the default risk is reduced. By linking this finding to previous findings, we would argue that when a CEO (probably also the founder of the firm) refrains from acting as a dictator and is surrounded by other people who share his or her vision and are involved with strategic planning, the CEO tenure has a positive effect on firm survival. The results further suggest that ownership concentration is positively related to the probability of default. Therefore, for SMEs, this may constitute a limit because it curtails the possibilities for development of the enterprise, and in a period of crisis, it may not allow for generation of adequate capital necessary for recovery. This is particularly true for family firms, which are known to be reluctant to expand the ownership structure in order to maintain control. Such a myopic vision could compromise firms’ survival and lead businesses towards bankruptcy. Finally, our empirical analysis highlighted that a higher percentage of women on boards reduces the default risk. The reason for this could be that women tend to communicate more effectively (Joy, 2008) and thus enhance dissemination of information from board to investors (Gul et al., 2011). Moreover, they allocate more time to monitoring and have a significant impact on board inputs by having better attendance records and joining more committees (Adams & Ferreira, 2009). Finally, the number of loans overdue by 180 days in the preceding 12 months and the number of months that firms are overdrawn – their cash and signature in the previous 12 months – are two key aspects which should be monitored with regard to a firm’s relationship with banks. Both are indicative of a firm’s inability to honor its debts on time and can be interpreted as a signal of inadequate management of working capital and financing sources.

Despite the interesting results, this paper does suffer from some limitations, which indicate new directions for future research. Firstly, we examined a limited number of years. It would therefore be interesting to replicate the complete analysis using a longer time horizon, in order to capture changes in corporate governance and bank-firm relationships that are unlikely to be able to save a firm on the verge of bankruptcy. Secondly, although we included three different categories of default determinants in our model, we did not have qualitative data, which could be collected through a survey of the field. Finally, even though we applied a robust technique, an interesting development for this research could be application of alternative techniques such as neural networks, support vector machines (SVMs) and machine learning models (Jones et al., 2015, 2017).

## References

[CR1] Adams R, Ferreira D (2009). Women in the boardroom and their impact on governance and performance. Journal of Financial Economics.

[CR2] Alaka HA, Oyedele LO, Owolabi HA, Kumar V, Ajayi SO, Akinade OO, Bilal M (2018). Systematic review of bankruptcy prediction models: Towards a framework for tool selection. Expert Systems with Applications.

[CR3] Altman EI (1968). Financial ratios, discriminant analysis and the prediction of corporate bankruptcy. The Journal of Finance.

[CR4] Altman EI, Giannozzi A, Roggi O, Sabato G (2013). Building SME rating: Is it necessary for lenders to monitor financial statements of the borrowers?. Bancaria.

[CR5] Angelini E, di Tollo G, Roli A (2008). A neural network approach for credit risk evaluation. The Quarterly Review of Economics and Finance.

[CR6] Bank of Italy. (2019). The 2018 Annual Report. Bank of Italy, May 31. Available at: https://www.bancaditalia.it/pubblicazioni/relazione-annuale/2018/

[CR7] Bartoli F, Ferri G, Murro P, Rotondi Z (2013). Bank–firm relations and the role of Mutual Guarantee Institutions at the peak of the crisis. Journal of Financial Stability.

[CR8] Beaver WH (1966). Financial ratios as predictors of failure. Journal of Accounting Research.

[CR9] Berger AN, Frame WS, Miller N (2005). Credit scoring and the availability, price and risk of small business credit. Journal of Money, Credit, and Banking.

[CR10] Berger A, Udell G (1995). Relationship lending and lines of credit in small business finance. Journal of Business.

[CR11] Bergerès AS, d’Astous P, Dionne G (2015). Is there any dependence between consumer credit line utilization and default probability on a term loan? Evidence from bank-customer data. Journal of Empirical Finance.

[CR12] Bernardo, J., & Smith, A. (1994). *Bayesian theory*. Wiley.

[CR13] Bottazzi G, Grazzi M, Secchi A, Tamagni F (2011). Financial and economic determinants of firm default. Journal of Evolutionary Economics.

[CR14] Calabrese R, Marra G, Osmetti SA (2013). Bankruptcy prediction of small and medium enterprises using a flexible binary generalized extreme value model. Journal of Applied Statistics.

[CR15] Caliendo M, Kopeinig S (2008). Some practical guidance for the implementation of propensity score matching. Journal of Economic Surveys.

[CR16] Carcello JV, Neal TL (2003). Audit committee independence and disclosures: Choice for financially distressed firms. Corporate Governance: An International Review.

[CR17] Chatterjee, S., & Hadi, A. S. (2012). *Regression analysis by example* (5th ed.). Wiley.

[CR18] Chen Y, Huang RJ, Tsai J, Tzeng LY (2015). Soft information and small business lending. Journal of Financial Services Research.

[CR19] Chi G, Meng B (2019). Debt rating model based on default identification. Management Decision.

[CR20] Christopoulos AG, Dokas IG, Kalantonis P, Koukkou T (2019). Investigation of financial distress with a dynamic logit based on the linkage between liquidity and profitability status of listed firms. Journal of the Operational Research Society.

[CR21] Ciampi, F. (2015). Corporate governance characteristics and default prediction modeling for small enterprises. An empirical analysis of Italian firms. *Journal of Business Research,**68*(5), 1012–1025.

[CR22] Ciampi, F., Giannozzi, A., Marzi, G., & Altman, E. I. (2021). Rethinking SME default prediction: a systematic literature review and future perspectives. *Scientometrics*, 1–48. 10.1007/s11192-020-03856-0 PMC7844786 33531720

[CR23] Ciampi F (2018). Using corporate social responsibility orientation characteristics for small enterprise default prediction. WSEAS Transactions on Business and Economics.

[CR24] Ciampi F, Gordini N (2013). Small enterprise default prediction modeling through artificial neural networks: An empirical analysis of italian small enterprises. Journal of Small Business Management.

[CR25] Dainelli F, Giunta F, Cipollini F (2013). Determinants of SME credit worthiness under Basel rules: The value of credit history information. PSL Quarterly Review.

[CR26] Dietsch M, Petey J (2002). The credit risk in SME loans portfolios: Modeling issues, pricing, and capital requirements. Journal of Banking & Finance.

[CR27] Dietsch M, Petey J (2004). Should SME exposures be treated as retail or corporate exposures? A comparative analysis of default probabilities and asset correlations in French and German SMEs. Journal of Banking & Finance.

[CR28] Duarte FD, Gama APM, Gulamhussen MA (2018). Defaults in bank loans to SMEs during the financial crisis. Small Business Economics.

[CR30] Edmister RO (1972). An empirical test of financial ratio analysis for small business failure prediction. Journal of Financial and Quantitative Analysis.

[CR31] El Kalak I, Hudson R (2016). The effect of size on the failure probabilities of SMEs: An empirical study on the US market using discrete hazard model. International Review of Financial Analysis.

[CR32] Elsas R (2005). Empirical determinants of relationship lending. Journal of Financial Intermediation.

[CR33] Fantazzini D, Figini S (2009). Default forecasting for small-medium enterprises: Does heterogeneity matter?. International Journal of Risk Assessment and Management.

[CR34] Fidrmuc J, Hainz C (2010). Default rates in the loan market for SMEs: Evidence from Slovakia. Economic Systems.

[CR35] Figini S, Giudici P (2011). Statistical merging of rating models. Journal of the Operational Research Society.

[CR36] Filipe SF, Grammatikos T, Michala D (2016). Forecasting distress in European SME portfolios. Journal of Banking & Finance.

[CR37] Gul F, Srinidhi B, Ng A (2011). Does board gender diversity improve the informativeness of stock prices?. Journal of Accounting and Economics.

[CR38] Gupta J, Gregoriou A, Ebrahimi T (2018). Empirical comparison of hazard models in predicting SMEs’ failure. Quantitative Finance.

[CR39] Gupta J, Gregoriou A, Healy J (2015). Forecasting bankruptcy for SMEs using hazard function: To what extent does size matter?. Review of Quantitative Finance and Accounting.

[CR40] Hasumi R, Hirata H (2014). Small Business Credit Scoring and Its Pitfalls: Evidence from Japan. Journal of Small Business Management.

[CR41] Hay CD, Baskerville FR, Qiu T (2007). The association between partnership financial integration and risky audit client portfolios. Auditing.

[CR42] Heckman JJ, Ichimura H, Todd P (1998). Matching as an econometric evaluation estimator. The Review of Economic Studies.

[CR43] Jones, S., Johnstone, D., & Wilson, R. (2017). Predicting corporate bankruptcy: An evaluation of alternative statistical frameworks. *Journal of Business Finance & Accounting,**44*(1) & (2), 3–34.

[CR44] Jones S, Johnstone D, Wilson R (2015). An empirical evaluation of the performance of binary classifiers in the prediction of credit ratings changes. Journal of Banking & Finance.

[CR45] Joy, L. (2008). Women board directors in the United States: An eleven-year retrospective. In S. Vinnicombe, V. Singh, R. J. Burke, D. Bilimoria, & M. Huse (Eds.), *Women on corporate boards of directors* (pp. 15–23). Edward Elgar Publishing Inc.

[CR46] Kanapickiene R, Spicas R (2019). Credit risk assessment model for small and micro-enterprises: The case of Lithuania. Risks.

[CR47] Keasey K, Watson R (1987). Non-financial symptoms and the prediction of small company failure: A test of Argenti’s Hypotheses. Journal of Business Finance & Accounting.

[CR48] Keasey K, Watson R (1988). The non-submission of accounts and small company financial failure prediction. Accounting and Business Research.

[CR49] Kosmidis K, Stavropoulos A (2014). Corporate failure diagnosis in SMEs. International Journal of Accounting and Information Management.

[CR50] Lin FY, Liang D, Chu WS (2010). The role of non-financial features related to corporate governance in business crisis prediction. Journal of Marine Science and Technology.

[CR51] Lin S-M, Ansell J, Andreeva G (2012). Predicting default of a small business using different definitions of financial distress. Journal of the Operational Research Society.

[CR52] McKillop D, French D, Quinn B, Sobiech AL, Wilson JOS (2020). Cooperative financial institutions: A review of the literature. International Review of Financial Analysis.

[CR53] Merton RC (1974). On the pricing of corporate debt: The risk structure of interest rates. The Journal of Finance.

[CR54] Migliani S, Kamran A, Darren H (2015). Voluntary corporate governance structure and financial distress: Evidence from Australia. Journal of Contemporary Accounting & Economics.

[CR55] Mittal S, Gupta P, Jain K (2011). Neural network credit scoring model for micro enterprise financing in India. Qualitative Research in Financial Markets.

[CR56] Nam C (2013). Dynamic credit risk model for SMEs. Asian Review of Financial Research.

[CR57] Norden L, Weber M (2010). Credit line usage, checking account activity, and default risk of bank borrowers. The Review of Financial Studies.

[CR58] Ohlson JA (1980). Financial ratios and the probabilistic prediction of bankruptcy. Journal of Accounting Research.

[CR59] Oliveira MDNT, Ferreira FAF, Ilander GOP-B, Jalali MS (2017). Integrating cognitive mapping and MCDA for bankruptcy prediction in small- and medium-sized enterprises. Journal of the Operational Research Society.

[CR60] Ono A, Hasumi R, Hirata H (2014). Differentiated use of small business credit scoring by relationship lenders and transactional lenders: Evidence from firm–bank matched data in Japan. Journal of Banking & Finance.

[CR61] Petersen MA, Rajan RG (1994). The benefits of lending relationships: Evidence from small business data. Journal of Finance.

[CR62] Pompe PM, Bilderbeek J (2005). The prediction of bankruptcy of small- and medium-sized industrial firms. Journal of Business Venturing.

[CR63] Rosenbaum PR, Rubin DB (1983). The central role of the propensity score in observational studies for causal effects. Biometrika.

[CR64] Saurina J, Trucharte C (2004). The impact of Basel II on lending to small-and medium-sized firms: A regulatory policy assessment based on Spanish credit register data. Journal of Financial Services Research.

[CR65] Shin KS, Lee TS, Kim HJ (2005). An application of support vector machines in bankruptcy prediction model. Expert Systems with Applications.

[CR66] Shin KS, Lee YJ (2002). A genetic algorithm application in bankruptcy prediction modeling. Expert Systems with Applications.

[CR67] Smondel A (2018). SMEs’ soft information and credit rationing in France. Human Systems Management.

[CR68] Sohn SY, Jeon H (2010). Competing risk model for technology credit fund for small and medium-sized enterprises. Journal of Small Business Management.

[CR69] Sohn SY, Kim YS (2013). Behavioral credit scoring model for technology-based firms that considers uncertain financial ratios obtained from relationship banking. Small Business Economics.

[CR70] Traczynski J (2017). Firm default prediction: A bayesian model-averaging approach. Journal of Financial and Quantitative Analysis.

[CR71] Yazdanfar D (2011). Predicting bankruptcy among SMEs: Evidence from Swedish firm-level data. International Journal of Entrepreneurship and Small Business.

[CR72] Yildirim, A. (2020). The effect of relationship banking on firm efficiency and default risk. *Journal of Corporate Finance,**65*, 101500.

[CR73] Yu S, Chi G, Jiang X (2019). Credit rating system for small businesses using the K-S test to select an indicator system. Management Decision.

[CR74] Zmijewski ME (1984). Methodological issues related to the estimation of financial distress prediction models. Journal of Accounting Research.

